# Count-rate management in ^131^I SPECT/CT calibration

**DOI:** 10.1186/s40658-025-00718-7

**Published:** 2025-01-26

**Authors:** Staffan Jacobsson Svärd, Cecilia Hindorf, Joachim N. Nilsson

**Affiliations:** 1 Department of Medical Radiation Physics and Nuclear Medicine, Karolinska University Hospital, Solna, Sweden; 2 Department of Molecular Medicine and Surgery, Karolinska Institutet, Stockholm, Sweden

**Keywords:** SPECT/CT, ^131^I, Quantification, Calibration, Dosimetry

## Abstract

**Background:**

System calibration is essential for accurate SPECT/CT dosimetry. However, count losses due to dead time and pulse pileup may cause calibration errors, in particular for ^131^I, where high count rates may be encountered. Calibration at low count rates should also be avoided to minimise detrimental effects from e.g. background counts and statistical fluctuations. This paper aims to present experimental data illustrating count-rate dependencies and to propose practical routines to mitigate errors in the ^131^I calibration procedure without needing advanced analysis tools.

**Results:**

The sensitivities of two General Electric (GE) Discovery 670 Pro systems were assessed using two Jaszczak phantom geometries. SPECT/CT data were collected over two months, starting with an initial ^131^I content of > 2 GBq, decaying to approximately 20 MBq. This allowed for a detailed analysis of count losses due to dead time and pulse pileup. From the sensitivity analysis, it was shown that robust calibration was obtained for ^131^I phantom activities ranging between 250 and 1500 MBq.

**Conclusions:**

The results show that adequate corrections for dead-time and pulse-pileup counting losses are essential for accurate calibration. It is argued that loss corrections should be based on total spectrum count rates in projections and not only on the 364.5 keV energy window data. The measurement campaigns presented in this paper, using basic tools and equipment, may serve as a model for establishing routines for count-loss corrections as well as for system calibration and regular control of system sensitivity. The data suggest that analysis of source and count concentration in a homogeneous Jaszczak phantom offers robust calibration, whereas analysis of source strength and counts in a delineated phantom insert offers a practical and robust method for regular quality control.

## Introduction

Dosimetry for radionuclide therapy has enjoyed a steadily increasing interest and demand over the latest decades, which has led to a set of publications with guidelines for quantitative SPECT and dosimetry [1–5]. A central procedure common to these guidelines is proper calibration of the SPECT system, which allows the reconstructed counts in an image stack to be directly transferable to activity.

Although the guidelines give some directives for the calibration procedure, there is still a need for each nuclear medicine department to design their own calibration routines, for which accurate count-rate management may be crucial for obtaining reliable results. With that in mind, this paper aims to present experimental data illustrating count-rate dependencies and to propose practical routines to mitigate errors in the ^131^I calibration procedure. The routines are intended to be applicable for the general user, who has access to a clinical SPECT/CT system with general analysis and reconstruction software. The user should also have access to a standard cylindrical Jaszczak phantom, in accordance with guidelines for ^131^I mIBG dosimetry [4], preferably also with a 6 cm spherical insert. The intention is to present routines independent of special-purpose software for more advanced data analysis, or more advanced phantom geometries.

The strong gamma-ray emission of ^131^I may give rise to high count rates and associated count losses already at low amounts of activity relative to what is administered in clinical radiotherapy. In this study, 10% count loss was encountered at a phantom ^131^I activity of about 250 MBq. The need for count-loss corrections in clinical ^131^I quantification has been long acknowledged, e.g. in planar imaging [6]. Recent guidelines for ^131^I mIBG dosimetry [4] emphasise the need for adequate count-loss corrections, and the challenges encountered with respect to count-loss corrections in SPECT/CT calibration for multicentre ^131^I dosimetry studies are further illustrated in [7].

At high count rates, two primary types of count losses must be managed: dead time and pulse pileup [8]. Dead-time losses are related to data-acquisition electronics being busy processing one pulse when the next arrives, a situation resulting in the rejection of the second pulse. Pulse-pileup losses occur when pulses overlap within the data acquisition system’s time frame, rendering them indistinguishable. While dead time losses are frequently discussed and corrected, pulse pileup losses are often overlooked or mistakenly combined with dead time losses.

Dead time losses can be managed based on system characteristics. The data acquisition system may either be paralysable, non-paralysable or a combination of the two, leading to different mathematical expressions for the losses [8]. For a paralysable system, the spectrum count rate will pass a maximum and then decrease as stronger sources are placed in front of the detector. The spectrum count rate in a non-paralysable system will steadily rise towards a finite, asymptotic value as higher activities are presented. Accurate corrections for dead-time losses can be introduced based on total spectrum count rate, provided that (i) the count rate is not exceedingly high (losses greater than 30–40% should be avoided [8]), and (ii) the energy spectrum covers a majority of the pulse amplitudes entering the system. For the systems in this study, 30% dead-time losses were estimated at a total spectrum count rate of 156 kcps.

While dead-time losses appear already at relatively low count rates (in this study, 1% dead time was estimated at a total spectrum count rate of about 6.2 kcps), losses from pulse pileup are expected to become significant at much higher rates, causing losses more challenging to manage. In this case, two pulses (or more) entering simultaneously are summed and thus end up in a different part of the energy spectrum, both being lost from their respective original energy channel. In addition, the pileup event leads to an underestimation of the total count rate, because the two pulses are counted as one. The summed amplitude may even become higher than the spectrum limit, implying that both pulses may be lost, and the total count rate may be further underestimated. Since pileup losses depend on the properties of the energy spectrum and the energy window settings for the nuclide being studied, these losses will vary between different radionuclides and between examinations. Factors such as the level of scattering, which is dependent on the subject being imaged, can further influence pileup losses.

In the calibration procedure, accurate management of all count losses are important. In addition, systematic errors may also arise at low activities, where results may be influenced by background counts, by large statistical fluctuations or by how reconstruction software handles low-level statistics.

In this article, it is suggested that calibration is based on a series of Jaszczak phantom measurements, covering a wide range of count rates while allowing for development of routines for count-loss management. The presented methodology may also be applicable for developing count-loss-correction schemes for clinical examinations, a topic which is beyond the scope of this study.

## Materials and methods

### Activity meter and source assessment

SPECT/CT calibration requires samples with well-known activity. Accordingly, having access to an activity meter that is traceable to a national or international standard is crucial for accurate calibration. In this study, a Veenstra VDC-505 m (Veenstra, Joure, The Netherlands) was used to assess the samples used. This meter undergoes regular quality control, including comparisons to standards. Since the scope of this study was count-rate management in calibration rather than system calibration itself, it was not considered necessary to use a ^131^I reference source for calibration close in time to the study. However, based on comparisons to traceable standards at other occasions, the accuracy in ^131^I measurements is estimated to about 2%. Stability controls performed daily over several years using a 7 MBq ^137^Cs source indicate a precision below 3%.

In this study, all sources were only measured once with the activity meter. Since accurate relative activity was considered most important for the aims of this study, it was selected to (i) base phantom activity on physical decay, and (ii) consistently use the same sources and phantoms for the two SPECT/CT systems.

### SPECT/CT systems

The data presented in this paper were collected with two GE Discovery 670 Pro SPECT/CT systems (GE Healthcare, Haifa, Israel), which exhibit the characteristics of a paralysable data acquisition system. According to personal communication with a GE representative^1^, front-end-electronics block pulses that are too close in time in order to prevent pile-up effects, efficiently manifesting as a dead time τ per detector pulse of 1.6 μs in the standard setting. However, pileup from two pulses close enough in time to be indistinguishable must still be expected despite this functionality.

The SPECT/CT measurements were performed using the High Energy General Purpose (HEGP) collimators, and the reconstructions were executed on a Xeleris workstation using the Volumetrix MI Evolution software. However, it is expected that the procedures presented in this paper are applicable to any SPECT/CT system and any reconstruction software.

### Energy settings

Because dead-time and pulse-pileup losses depend primarily on the total number of pulses in the detector, energy protocols were defined to cover the total available energy range, in addition to an ^131^I-specific triple-energy window (TEW) protocol. The energy protocols used are presented in Table 1.

**Table 1:** Energy protocols used in this work

Protocol name	Window type*	Energy range [keV]
Open_HE	EM	[0,681]
I131TEW	EM	364.5 ± 29.2 (8%)
	SC	317.5 ± 14.6
		411.5 ± 14.6

* Emission (EM) or scatter (SC)

The Discovery 670 Pro system’s “high energy” option was applied, widening the available energy range from the standard [0,512] keV to [0,681] keV, thus enabling the use of the “Open_HE” protocol.

### Planar measurements

#### Assessment of system dead time

The dead time per detector pulse was determined for each detector head of the two SPECT/CT systems using the two-source method [9], with the purpose to independently validate the manufacturer-given value. Two syringes were prepared with ^131^I activities of 49.2 and 49.9 MBq. These were placed in the point-source position assigned for control of intrinsic uniformity, more than 3 m from the surface of the detector. Measurements were performed without collimation, using the so-called intrinsic collimators, i.e. plastic covers that protect the detectors. The two ^131^I sources were measured separately and in combination, resulting in total spectrum count rates R_1_, R_2_ and R_12_, respectively, collected using the Open_HE energy protocol (see Table 1). The system dead time was given by Eq. (1), assuming a paralysable system:

1 $$\:\tau \: = \frac{{2 \cdot \:{R_{12}}}}{{{{\left( {{R_1} + {R_2}} \right)}^2}}} \cdot \:ln\left( {\frac{{{R_1} + {R_2}}}{{{R_{12}}}}} \right)$$

The response of a paralysable data acquisition system is defined by Eq. (2), describing the relation between the observed spectrum count rate, R_out_, and the true pulse rate, R_in_, depending on the dead time per pulse, τ.

2 $$\:{R_{out}} = {R_{in}} \cdot \:{e^{ - {R_{in}} \cdot \:\tau \:}}$$

To get the true number of detector events, a dead-time correction factor C_DT_ = R_in_/R_out_ must thus be applied to the measured number of counts. Because Eq. (2) does not allow an analytical solution for R_in_, a four-degree polynomial approximation P_DT_(R_out_) was adopted in this work to express C_DT_ as a function of R_out_. This is further described in the results section.

#### Assessment of count losses from dead time and pulse pileup

A set of intrinsic planar measurements, thus not using collimation, was performed for one detector head with the purpose of visualising how count losses from dead time and pulse pileup manifest at various count rates. For these measurements, four syringes were prepared with ^131^I activities of 25.0, 48.6, 51.9 and 88.2 MBq, totalling 213.7 MBq. These were placed more than 3 m from the surface of the detector one at the time and in various combinations. When used in combination, the syringes were placed > 2 cm apart to limit scattering.

Data were collected using both energy protocols in Table 1. Information on changes in the energy spectrum, expected due to pulse pileup, was extracted by analysing the ratio of counts in the emission and scattering windows of the 364.5 keV peak (EM/SC) and the ratio of scatter-corrected counts in the 364.5 keV window to the total spectrum counts ((EM-SC)/Open_HE).

In this study, the pileup losses in the 364.5 keV energy window were investigated, using the scatter-corrected counts (EMSC). The data were related to the total spectrum count rate, recorded using the Open_HE energy protocol, and corrected for dead-time losses using the polynomial P_DT_(R_out_) obtained from the dead-time assessment. Any remaining differences between measured and expected count rates were attributed to losses due to pulse pileup. In this procedure, known source contents were used while assigning correction factors C_PU_ to maintain constant (EM-SC) count rate per activity unit. For all data, background counts were subtracted, averaged from recordings without any source present before and after the source measurements. The time for all acquisitions was 60 s.

For presentation purposes, a third-degree polynomial fit P_PU_(R_out_) was made to the obtained C_PU_ data, which is further described in the results section. The uncertainties of the underlying data points are dominated by uncertainties in measured ^131^I activities, estimated to 2–3%. Uncertainties emanating from counting statistics were a magnitude smaller.

#### Assessment of extrinsic planar sensitivity

A basic ^131^I calibration measurement involves measuring the planar sensitivity using a point-like source or a Petri-dish source, albeit this type of calibration is claimed to be of doubtful use for SPECT/CT quantification [1, 3, 5]. In this study, extrinsic point-source planar-sensitivity measurements were performed for the purpose of obtaining a rough comparison with data obtained in SPECT/CT calibration. Three point sources (syringes) with a content of 12.3, 21.7 and 31.7 MBq ^131^I were placed consecutively between the two detectors, with the HEGP collimators mounted, and planar images were collected for 60 s.

Analyses of the sensitivity were performed using the I131TEW scatter corrected data (referred to as EM-SC), and total spectrum count rates were assessed using the Open_HE data (Table 1). The energy spectrum count rate was kept low enough to maintain a dead time of < 1.5%, although dead time corrections were applied. Background measurements were also performed and subtracted. Uncertainty estimates for the system sensitivities were calculated based on measurements of the three ^131^I sources and the two detectors of each system. In addition, the size of the image region subject for analysis was varied, allowing for analyses of its influence on measured sensitivity for the two systems.

### Tomographic phantom measurements

#### Objects under study

SPECT/CT measurements were performed on two phantoms at 11 occasions per phantom over a time period of two months, June-August and October-December 2022, respectively;

(A) A homogeneous water-filled Jaszczak phantom with an initial ^131^I activity of 2160 MBq and a final activity of 16 MBq;
(B) A cold water-filled Jaszczak phantom with a hot 6 cm diameter spherical insert, which was filled with an initial ^131^I activity of 2090 MBq and a final activity of 9 MBq.

To enable use of activity concentrations, the water volumes were assessed by measuring the weight before and after filling of phantoms as well as insert. Before adding ^131^I, potassium iodide was introduced to block the activity from attaching to the walls of the phantom and insert, respectively.

The long time of assessment enabled analyses over a wide range of system count rates. In this study, precision of relative activities was considered more important than the accuracy of absolute activities, governing the choice of repeated measurements over time, rather than filling the phantoms with gradually more activity. Whereas the accuracy of the absolute activities of the phantoms was limited by the accuracy of the activity meter, the uncertainties in relative activities in each set of phantom measurements were thus governed by physical decay only, leaving only a minor error from precision in the ^131^I half-life. Here a value of 8.0252 days was used [10].

#### Data collection and reconstruction

Both phantoms were measured using both SPECT/CT systems, producing a total of four data sets. All data were collected using clinical SPECT/CT protocols with the HEGP collimators and the I131TEW energy setting (see Table 1). The matrix size was 128 × 128 pixels, the auto-contour option was applied, and no zoom was used. Each SPECT data collection of phantom (A) comprised in total of 90 projections, collected for 8 s per projection, and was followed by CT data acquisition. Phantom (B) was assessed using 72 projections, collected for 25 s per projection. The change in the data collection scheme between phantoms was unintentional and occurred due to a change in clinical practice between the measurement series. The 2.5 times longer data collection for phantom (B) was reflected in a proportionally larger number of collected counts.

For phantom (A), data collection and analysis were repeated three times at each measurement occasion to deduce information on random uncertainties. The phantom was removed and replaced between each data collection. Keeping the data collection times constant over the measurement series implied a lower number of collected counts at lower phantom activities, thus larger random uncertainties were anticipated at lower activities. For phantom (B), no repetitions were made, motivated by previously recorded data showing that random uncertainty was subordinate to systematic differences emanating from variations in count rate.

In connection to every phantom measurement, the total spectrum count rate over a set of projection angles was also assessed using the Open_HE energy protocol (see Table 1). These data were collected to establish and utilise a relation between the average total spectrum count rate and counting losses in the 364.5 keV energy window due to dead time and pulse pileup. This procedure is further described below. Finally, background measurements were also collected, using an inactive Jaszczak phantom.

The reconstructions were executed using the clinic’s dedicated dosimetry reconstruction protocol, denoted as IRACSCRR in Xeleris. It is an OSEM iterative reconstruction (IR) scheme, applied with 10 subsets and 10 iterations, which adopts CT-based attenuation correction (AC), scattering correction (SC) and resolution recovery (RR). For comparison, some reconstructions were also executed when turning the resolution recovery option off, resulting in a reconstruction alternative denoted as IRACSC.

The series of measurements on phantom (A) were used to establish tomographic dead-time and pulse-pileup corrections as well as to perform a tomographic sensitivity calibration. The series of measurements on phantom (B) were used to control the applicability of the loss-correction scheme from (A) and to identify a robust procedure for regular calibration control.

#### Correcting for dead time and pulse pileup

In tomographic measurement and reconstruction, corrections for dead time and pulse pileup are more complicated than in planar measurements, because the projections are collected using two detector heads at various count rates. Consequently, accurate accounting for losses would require individual corrections for each projection. However, a typical contemporary SPECT/CT system does not provide the possibility to correct each projection individually, wherefore the user is left with the possibility to make a general correction to the complete data set. Alternatively, the user may develop more advanced self-build analysis tools, which falls beyond the scope of this work.

In this work, the following procedure was adopted for correcting tomographic ^131^I measurements for count losses;

i. Executing a series of SPECT/CT measurement of a homogeneous Jaszczak phantom with a range of known source contents of ^131^I, while collecting data using the Open_HE and the I131TEW energy protocols (see Table 1);
ii. Analysing the ratio between the total number of background-subtracted, scatter-corrected 364.5 keV energy window counts (EM-SC) in all projections and the ^131^I activity at the time of each SPECT acquisition;
iii. Defining a dead-time and pulse-pileup correction factor C_DTPU_(R_out_) for each data point, which returned a constant number of peak counts per activity unit, equal to the projected counts per MBq at the 0 MBq limit;
iv. Fitting a four-degree polynomial P_T_(R_out_) to the obtained C_DTPU_(R_out_) data, using the boundary condition P_T_(0) = 1. In this step, a subjectively selected limit in count rate was introduced to at R_out_ = 160 kcps, where about 50% losses were encountered;
v. Using the P_T_(R_out_) polynomial to correct the number of counts in reconstructed image stacks for dead-time and pulse-pileup losses based on the total spectrum count rate.

One may note that the correction scheme will only be valid up to the limit of R_out_ selected in the polynomial fit, which was 160 kcps in this work, and data points above this limit should be considered unreliable.

In this study, ratios of counts in the adopted energy windows were also used to provide information on changes in the energy spectrum due to pulse pileup. The adopted ratios were the same as in the planar measurements; (EM/SC), i.e. the ratio of counts in the emission and scatter windows of the 364.5 keV peak, and; ((EM-SC)/Open_HE), i.e. the ratio of scatter-corrected counts in the 364.5 keV window to the total spectrum counts.

#### Determining the SPECT/CT sensitivity

The reconstructed image stack may be used for calculating the SPECT/CT sensitivity using three methods;

- Using all voxel counts in the complete image stack, or full field of view (FOV), here called Full-FOV data, and calculating the sensitivity as the total number of counts per second per source unit [cps/MBq];
- Using the voxel-count concentration over a central region of the source and calculating the sensitivity by dividing with time and the source concentration [(cps/ml)/(MBq/ml)];
- Delineating the source in the image stack and calculating the sensitivity as the number of counts in the delineated object divided by time and its source content [cps/MBq].

Data from the homogeneous phantom (A) were used for determining the SPECT/CT system sensitivity. Calculation methods (a) and (b) were applied, which are expected to return similar values. Method (c) is expected to return a lower value due to imperfect spatial resolution and resulting partial-volume effects, and it was thus not used on data from phantom (A). However, this method is still relevant for regular calibration controls (see below).

The series of measurements allowed for assessment of how the level of phantom ^131^I activity influences the results. Whereas background counts may be considered negligible at moderate or high count rates, the scope of this study also included low count rates. Accordingly, all SPECT/CT data presented in this work included subtraction of background counts, which were obtained in measurements of an inactive, water-filled Jaszcak phantom. The background images were collected, reconstructed and analysed using the same settings as the active phantom data.

#### Verifying the calibration factor

Once the calibration factor (CF) has been determined, its consistence over time must be controlled regularly. In this article, such controls are suggested to be performed using phantom geometry (B), i.e. a Jaszczak phantom with a centrally placed spherical insert, which contains the activity to be assessed. The main advantages of using such a geometry are; (i) only the spherical insert is activated, leaving the Jaszczak phantom available for other quality control after the measurements, (ii) a spherical insert offers fast and practical delineation in reconstructed image stacks, and; (iii) as compared to other geometries with an insert or vial source, a central sphere provides data collected at fairly constant projection count rates, limiting errors that emanate from the lack of possibility to perform dead-time and pulse-pileup corrections on individual projections.

For the data collected using phantom geometry (B), all three sensitivity-calculation methods (a)(c) were used, allowing for assessment of how the level of ^131^I activity in the insert influences the results for each calculation method. One should bear in mind that method (c) will result in a lower sensitivity value because of partial-volume effects. Still, this method may be applicable for controlling the consistency of calibration over time.

## Results

### Planar measurements

#### Assessment of dead time

Dead time was assessed separately for both detectors of both SPECT systems. The spectrum count rates recorded with separate point sources were about 60 kcps, and with two sources together the count rates were about 100 kcps. Applying Eq. (1), values of τ between 1.60 μs and 1.68 μs were obtained for all four detectors, i.e. all values being within 5% from the manufacturer-given front-end-electronics setting of 1.6 μs per pulse. For one detector, the measurement was repeated, resulting in a value 3.5% from the first result. Considering the manufacturer-given value to be confirmed within the precision of these measurements, it was used for the further analyses below.

Using Eq. (2) and τ = 1.6 μs, R_in_ values were applied in steps of 1 kcps from 0 to 350 kcps to calculate corresponding R_out_ values and dead-time correction factors C_DT_. The maximum value of R_out_ was about 200 kcps. A fourth-degree polynomial fit P_DT_(R_out_) was then adopted as an analytic representation of C_DT_ as a function of R_out_, exhibiting a maximum deviation of < 1% from Eq. (2) over this range of count rates, which was considered adequate for the analyses below.

#### Assessment of count losses from dead time and pulse pileup

**Fig. 1:**
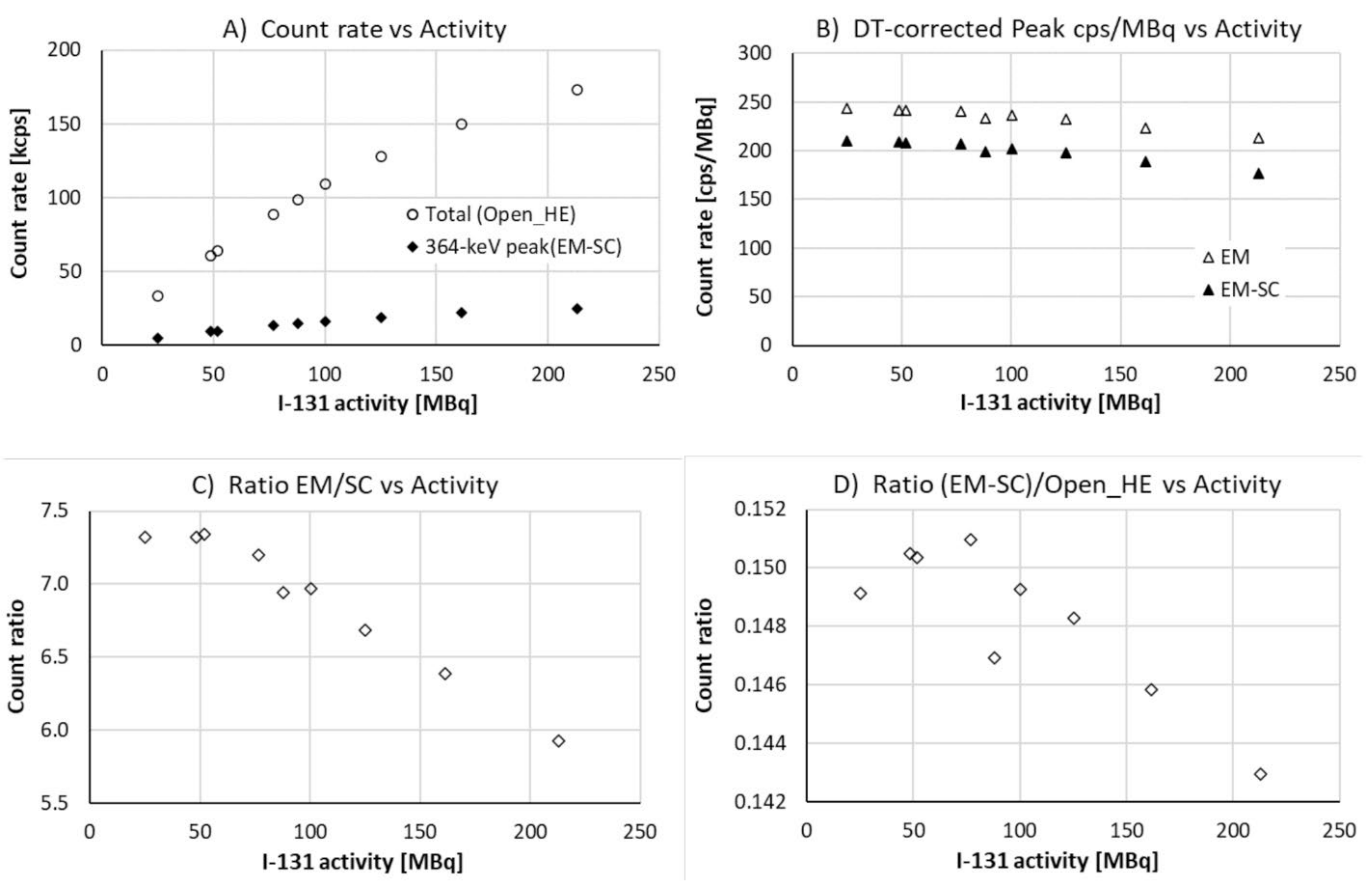
Data from planar measurements of ^131^I point sources using one detector head without collimation. (**A**) Total spectrum count rate (Open_HE) and scatter-corrected count rate in the 364.5 keV energy window (EM-SC). (**B**) Count rates per activity unit after correcting for dead-time count losses. (**C**) Ratio of emission and scattering counts in the 364.5 keV energy window (EM/SC). (**D**) Ratio of scatter-corrected counts in the 364.5 keV energy window to the total spectrum counts ((EM-SC)/Open_HE)

Additional planar measurements using the intrinsic collimator were executed for one detector head, to visualise how dead time and pulse pileup manifest at various count rates. Figure 1 presents data on recorded counts and count rates in the energy windows used (see Table 1).

As illustrated in Fig. 1A, count losses manifest as non-linear correlations between recorded count rates and ^131^I activity. Figure 1B shows that applying dead-time corrections (using the P_DT_(R_out_) polynomial derived in the previous section) is not sufficient to account for all losses, and changes in energy spectrum properties presented in Fig. 1 C and 1D provide evidence of pulse pileup effects.

**Fig. 2:**
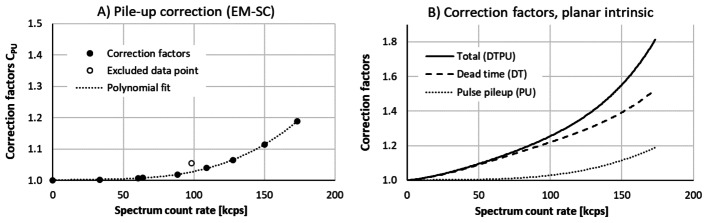
Correction factors obtained in measurements of ^131^I point sources using the intrinsic collimator. (**A**) Calculated pileup correction factors C_PU_. (**B**) All correction factors, illustrating how the total correction (DTPU) constitutes of corrections for dead time (DT) and pulse pileup (PU)

The data presented in Fig. 1B were used to calculate correction factors for pulse pileup (C_PU_) for each data point. These factors are presented in Fig. 2A, including a third-degree polynomial fit P_PU_(R_out_) to the C_PU_ data. In the fit, the constant term was set to unity, illustrated with a data point at R_out_ = 0. Figure 2B presents graphs of all correction factors, using the P_DT_ and P_PU_ polynomials and the total correction factor C_DTPU_ = P_DT_ ∙ P_PU_.

As illustrated in Fig. 2A, one data point is excluded from the polynomial fit to the calculated pileup correction factors C_PU_. This is justified by its deviating spectrum properties shown in Fig. 1D. The resulting total correction factor, presented in Fig. 2B, is dominated by dead time, but pulse pileup corrections are non-negligible at high count rates.

#### Assessment of extrinsic planar sensitivity

The planar sensitivity for ^131^I point sources using the HEGP collimator was measured and analysed for both detectors of the two SPECT/CT systems using three FOVs of different dimensions. For both systems and all FOV dimensions, the two detectors returned average planar sensitivities over the three separately measured ^131^I point sources within 0.13 cps/MBq from their common average, which was considered small enough for data from the two detectors to constitute common system data sets. Each calculated planar sensitivity was thus based upon six measurements, with results presented in Table 2.

**Table 2:** Planar system sensitivities in [cps/MBq], measured using HEGP collimators, ^131^I point sources and three different sizes of the analysed FOV

System	Full FOV (54 × 40 cm)	Circular FOV with 13.2 cm radius	Circular FOV with 6.6 cm radius
1	31.55 ± 0.24	30.16 ± 0.21	27.06 ± 0.20
2	30.33 ± 0.12	29.61 ± 0.15	27.18 ± 0.17

As shown in Table 2, the two systems exhibit equal sensitivity when limiting the analysed FOV to a circle with 6.6 cm radius, whereas system (1) exhibits higher sensitivity than system (2) when analysing the larger FOVs. The 4% difference between the two systems in full-FOV data may e.g. indicate unexpected contributions from peripheral parts of the detectors or more septum penetration for system (1). No evidence of extraneous sources was detected in the background measurements.

### Tomographic phantom measurements

#### Corrections for dead time and pulse pileup

**Fig. 3:**
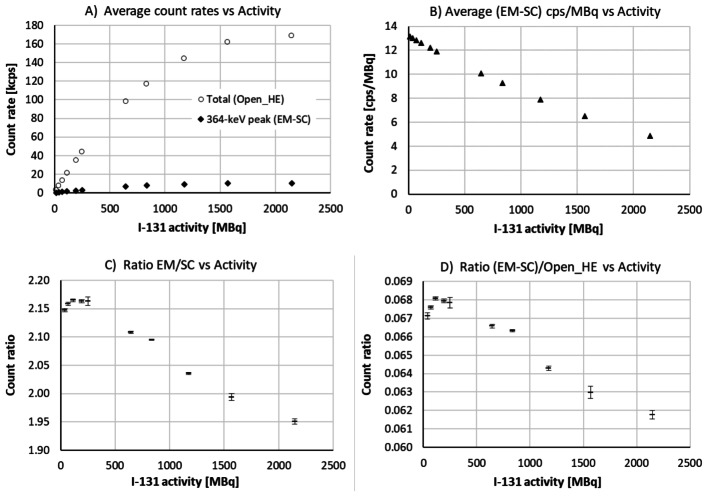
Projection data from tomographic measurements of a homogeneous Jaszczak phantom with ^131^I activity ranging from 16 MBq to 2160 MBq. (**A**) Total spectrum count rate (Open_HE) and scatter-corrected count rate in the 364.5 keV energy window (EM-SC), averaged over all recorded projections. (**B**) Average scatter-corrected count rate (EM-SC) per activity unit. (**C**) Ratio of emission and scattering counts in the 364.5 keV energy window (EM/SC). (**D**) Ratio of scatter-corrected counts in the 364.5 keV energy window to the total spectrum counts ((EM-SC)/Open_HE). The two latter graphs include error bars, presenting the standard deviations obtained in three consecutive measurements

Projection data collected in measurements of the homogeneous Jaszczak phantom (A) were used to establish a connection between losses of counts in the 364.5 keV energy window to the total spectrum count rate, using steps (i)-(iv) of the procedure described above. In addition, the ratios of counts in the adopted energy windows (see Table 1) were used to study how the energy spectrum changes with the count rate. The results for SPECT/CT system (1) are presented in Fig. 3. Almost identical results were obtained for SPECT/CT system (2).

As shown in Fig. 3A and B, average spectrum count rates of almost 170 kcps lead to > 60% count losses for the highest phantom activities. The losses are manifested in a steadily decreasing number of peak counts (EM-SC) per source unit with increasing phantom activities. Similar to the planar point-source data in Fig. 1 C and 1D, Fig. 3 C and 3D provide evidence of pulse-pileup effects, with changing energy-spectrum properties as the phantom activity increases.

**Fig. 4:**
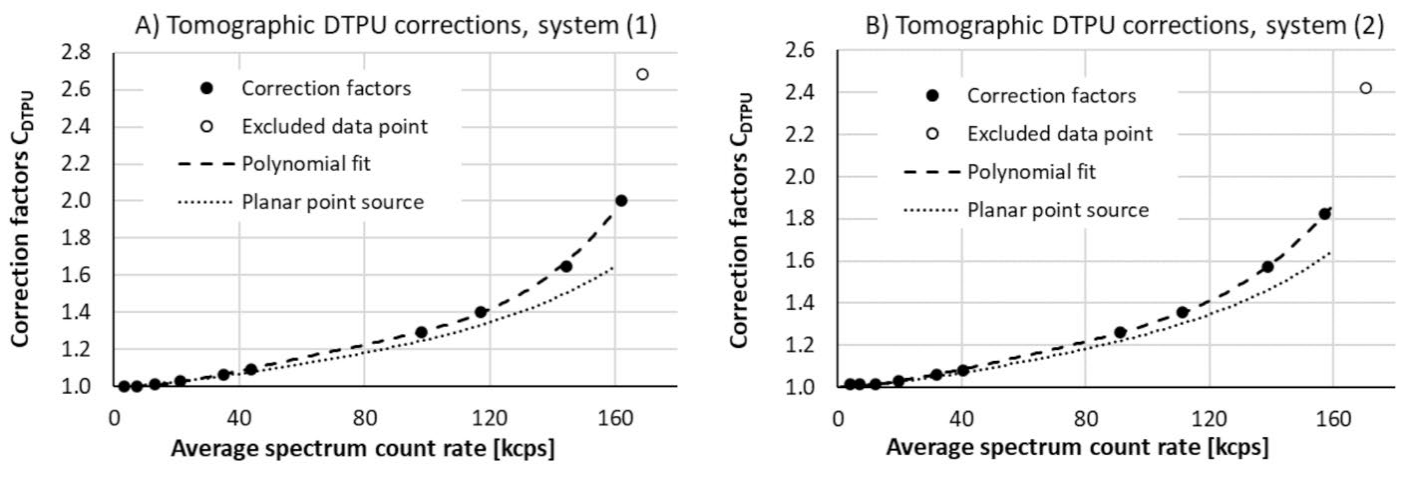
The dead-time and pulse-pileup (DTPU) loss-correction factors for the two systems (**A** and **B** shows the two systems separately), resulting from steps (iii-iv) in the presented procedure used for establishing corrections to losses in SPECT/CT measurements. The corresponding loss-correction polynomial obtained in the point-source measurements without collimation are presented for comparison. For both systems, the data point with the highest spectrum count rate was excluded from the fit, based on a decision to limit further analyses to spectrum count rates below 160 kcps

The resulting dead-time and pulse-pileup (DTPU) loss correcting factors for the two systems are presented in Fig. 4. including the polynomials P_T_(R_out_) fitted in step (iv). The corresponding polynomials obtained in planar point-source measurements without collimation are included for comparison. It was decided to exclude the highest data point for each system from the polynomial fit and limit further analyses to spectrum count rates below 160 kcps. The reason for this choice was the strong gradient of DTPU-correction values above 160 kcps, causing unacceptably high sensitivity to measured spectrum count rate at higher count rates.

As compared to the point-source measurements without collimation, a much smaller fraction of the collected events belongs in the 364.5 keV energy window (see Figs. 1D and 3D). This may be attributed to scattering in the Jaszczak phantom and in the collimators, leading to counts at lower energies. Consequently, one may expect pulse pileup from scattered events to be more severe, which may explain the need for higher correction factors in the phantom measurements, as illustrated in Fig. 4. As further discussed below, one may also expect a need for higher DTPU correction factors to averaged SPECT data due to variations in count rates between projections.

### Tomographic sensitivity as a function of source content

**Fig. 5:**
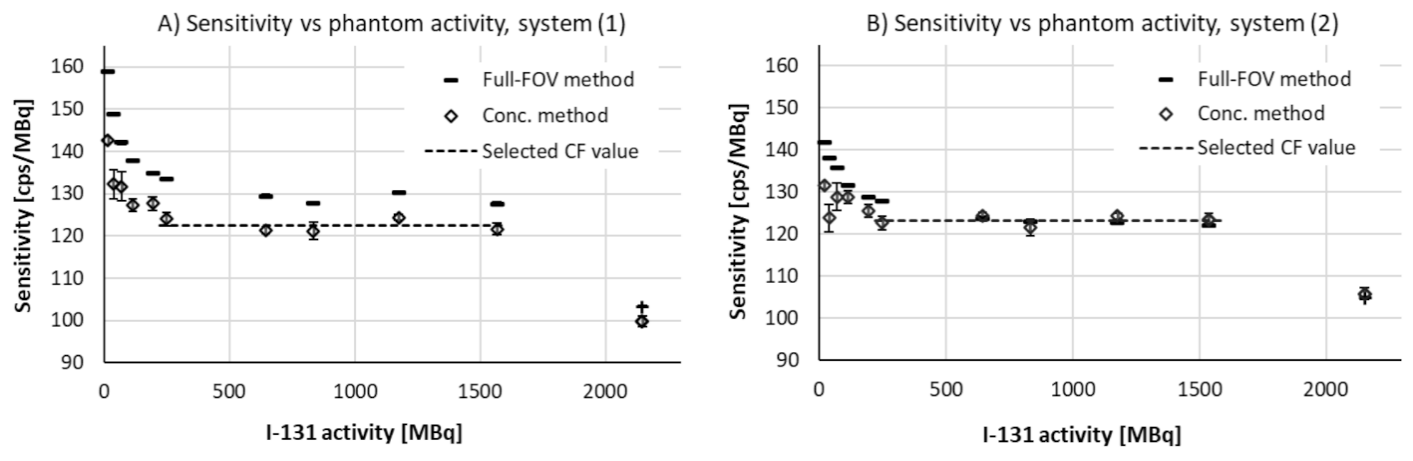
Sensitivity of the two SPECT/CT systems under study (**A** and **B** shows the two systems separately), obtained in measurements on a homogeneous Jaszczak phantom with ^131^I activity ranging from 16 MBq to 2160 MBq. The sensitivity was relatively stable at a phantom activity ranging between about 250 and 1600 MBq, and the resulting value of the calibration factor (CF) was selected as the average for the concentration method over this range

The reconstructed image stacks were used to calculate system sensitivity using the fullFOV method (a) and the concentration method (b). The data were corrected for dead time and pulse pileup, based on the total spectrum count rate R_out_ and the fitted polynomials P_T_(R_out_) described above. The resulting sensitivities for the two SPECT/CT systems are presented in Fig. 5.

As shown in Fig. 5, the measured sensitivities were relatively stable over a phantom activity ranging between about 250 and 1600 MBq. The deviating values at the highest activity can be attributed to insufficient DTPU correction, which is expected because the count rate exceeded the maximum accepted rate of 160 kcps defined when performing the polynomial fits. The deviating results at low activities are more difficult to explain but have been identified also in previous work on SPECT quantification, albeit for ^177^Lu phantoms 11, 12]. Likely reasons subject for discussion were inadequate scatter correction [11] and influence of background [12]. It can be noted that the total spectrum count rate in the lower part of the stable range (250 MBq ^131^I) in this study was about 40 kcps, implying a DTPU correction of about 9–10%, of which the pileup correction contributes with 2–3% points.

In theory, one may expect the full-FOV method and the concentration method to return similar results for objects of sufficient size, such as the homogeneous Jaszczak phantom. This proved valid for system (2), but not for system (1), where 6% more counts were collected in the full-FOV data. An enhanced number of counts in the detector periphery was indicated for system (1) also in the planar extrinsic measurements. Possible reasons are unexpected contributions for the peripheral detector parts or more septum penetration. Image uniformity was controlled and did not indicate any errors.

In this work, the results for the concentration method were used when selecting the resulting sensitivity values for three reasons; (i) data central in the image stack are less prone to influence from extraneous error sources; (ii) the detector periphery is excluded, like it is for dosimetric data, and; (iii) the deviations at low phantom activities were found to be less severe. The resulting sensitivities, selected as the average between 250 and 1600 MBq were 122.5 cps/MBq for system (1) and 123.3 cps/MBq for system (2).

Finally, one may note that the sensitivity values obtained from SPECT/CT data were about four times higher than the values obtained in planar sensitivity measurements. This was expected because of a default vendor setting, multiplying data by a factor of four when applying the resolution recovery reconstruction option. For comparison, IRACSC reconstructions were also performed, i.e. turning the resolution recovery off. These reconstructions returned sensitivity values slightly below 30 cps/MBq. However, the clinical dosimetry protocol applies IRACSCRR reconstruction, and consequently the selected sensitivity values were based upon IRACSCRR data.

### Verifying the system calibration

**Fig. 6:**
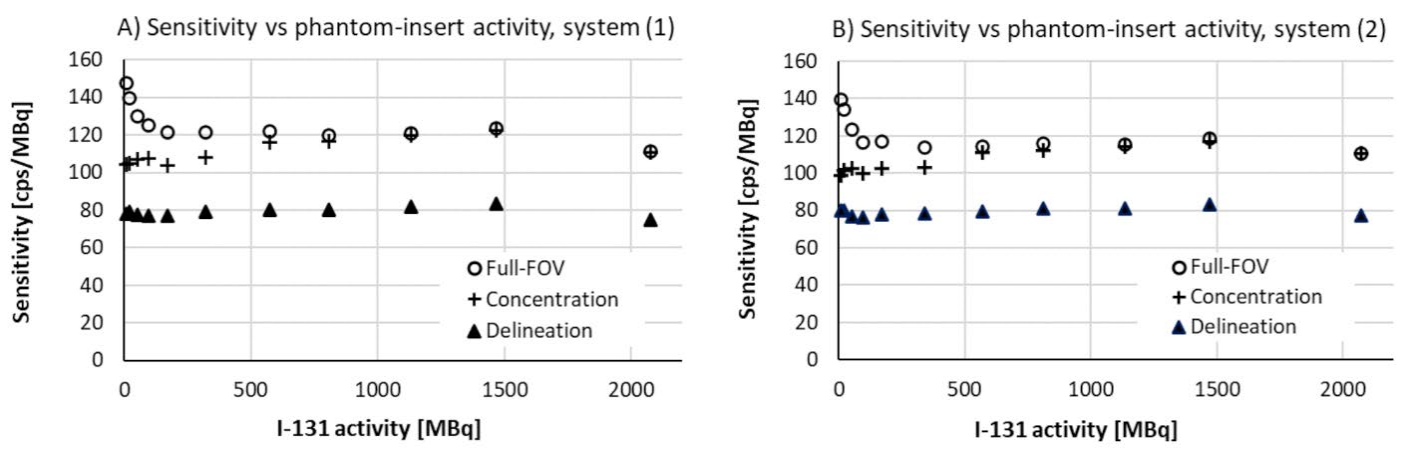
Verification of the system calibration using a Jaszczak phantom with a 6 cm spherical insert with ^131^I activity ranging from 9 MBq to 2070 MBq. In the figure, **A** and **B** shows the two systems separately. Both systems under study present inconsistencies at the highest as well as the lowest phantom activities

Once established, the system calibration should be verified regularly. In this study, the applicability of phantom (B) for such calibration control has been investigated. Measuring a range of phantom activities allowed for; (i) controlling the validity of the DTPU correction method using average total spectrum count rate, and (ii) examining the consistency of the sensitivity calculation methods (a)(c). The results of these measurements are presented in Fig. 6.

Figure 6 presents stable results for all methods at phantom-insert activities between 500 and 1200MBq and some inconsistencies at lower and higher activities. The deviations at higher activities, being < 8% from average in the [500,1200] MBq range for all three methods and both systems, were expected, because the count rate exceeded 170 kcps and the DTPU corrections were only defined for rates below 160 kcps. As the activity approaches zero, the fullFOV method and the concentration method exhibit relatively strong opposite deviations, going toward maximum 22% higher and 12% lower values, respectively. The delineation method offers the most robust data with an overall maximum deviation of 7% for the two systems, albeit as expected at a lower level due to partial-volume effects. The random uncertainty was not assessed, but as compared to the homogeneous Jaszczak phantom measurements, longer data collection resulted in more than twice the number of collected counts at the same activity. Accordingly, one may assume smaller uncertainties for the data in Fig. 6 as compared to the error bars presented in Fig. 5.

It was considered whether camera instability could be the reason for deviations at low phantom activity. However, similar behaviour of the two systems as well as opposite deviations for the full-FOV and concentration methods indicate other reasons. Acknowledging that accurate quantification at low activities is crucial in dosimetry, identifying the underlying causes of these deviations and mitigating them fall beyond scope of this work and should be subject for further studies.

Based on the results in Fig. 6, regular controls of system calibration for ^131^I are suggested to be performed using phantom geometry (B) and evaluated using the delineation method. ^131^I activities between 250 and 1500 MBq in the 6 cm spherical insert gave robust results, in accordance with the results for the homogeneous phantom (A). However, the lower part of this activity range would be recommended for regular control to limit dose to personnel. In this study, three data points were recorded for each system at phantom activities between 250 and 1000 MBq, resulting in average sensitivity values for the two systems of 79.9 cps/MBq and 79.8 cps/MBq, with a maximum deviation below 2% in any of the six data points.

## Discussion

### Methodology for dead-time and pulse pileup correction

In this article, count-loss corrections are proposed to be based on total spectrum count rate in recorded projections, being the driving force for both dead-time and pulse-pileup losses. Previous research [13, 14] also suggests that basing loss corrections on total spectrum count rate is robust for altering object geometries. However, it requires the collection of total spectrum data, in addition to the ^131^I peak data. In this work, the additional data collection with the Open_HE energy protocol comprised fewer angles and shorter time per angle, prolonging the total measurement time only by 2–3 min, which may be considered an acceptable cost for mitigating systematic errors. Alternatively, multiple energy windows may be used to keep track of total spectrum count rates.

An alternative loss-correction approach used e.g. in [11] utilises only the recorded peak count rate, assuming it to be representative for total count rate. However, the peak-to-total count ratio will depend on object properties; (i) a larger object/central source produces more scattered events than a smaller object/peripheral source, and; (ii) an object with high activity causes more pulse pileup than an object with low activity. The latter effect is e.g. illustrated in Fig. 3D, whereas the former effect can be demonstrated by comparing data from the two Jaszczak phantom geometries measured in this work. Despite relatively similar geometries, the ratios differed by about 15% for the emission window [EM/Open_HE] and about 25% for scatter-subtracted peak data [(EMSC)/Open_HE]. Notable is that a systematic 15% error in estimated count rate would cause a systematic 20–30% error in DTPU correction at a total spectrum count rate of about 150 kcps.

Because pulse-pileup losses depend on gamma-ray-spectrum properties, one must expect some remaining object dependence, even when basing the DTPU corrections on total spectrum count rate. Accordingly, a scheme for DTPU corrections should preferably be developed using a geometry representing the object under study. In this work, a correction scheme was developed for a cylindrical Jaszczak phantom. A similar scheme for clinical patient examinations may be developed by adopting the methodologies presented in this article but using e.g. an anthropomorphic phantom. This falls, however, beyond scope of this study.

Another problem is the absence of built-in tools for making count-loss corrections on individual projections, which is further discussed in the outlook section below. The measurements presented in this study adopt the use of Jaszczak phantoms with homogeneous activity, respectively with activity in a central sphere. Both these geometries offer the advantage of relatively small variations in projection count rates. Errors emanating from a lack of possibility to make individual loss corrections on each projection separately are thus minimised.

### Robust routines for establishing and verifying system calibration

This work has presented a set of measurements, which may serve as a template for robust SPECT/CT calibration routines for ^131^I;

1. Executing a set of calibration measurements of a homogeneous Jaszczak phantom, starting with a ^131^I activity of at least 2 GBq and continuing over a time span of about 2 months. (Alternatively, small quantities of well-known activity can be continually added to the phantom, [7].)
  The data should be recorded using clinical SPECT/CT protocols for ^131^I dosimetry.
  A methodology for DTPU corrections should be developed based on projection count rates, making use of the well-known ^131^I content at each time point, being governed solely by physical decay.
  Image stacks should be reconstructed using clinical protocols for ^131^I dosimetry.
  The system sensitivity at each source strength should be determined based on reconstructed image stacks, while deploying DTPU corrections.
  The resulting sensitivity should be selected as the average value over a range of phantom activities where it is proven to be stable.
2. Executing a set of verification measurements, using a Jaszczak phantom with a spherical ^131^I-activated insert, starting with a ^131^I activity of at least 2 GBq and continuing over a time span of about 2 months.
  The dimensions of the spherical insert should preferably be large enough to limit partial-volume effects in the insert centre.
  Data should be recorded, and image stacks should be reconstructed using clinical SPECT/CT protocols for ^131^I dosimetry.
  The image data should be subject to DTPU corrections, using projection count rates and the methodology developed under point 1 above.
  A range of ^131^I activities should be identified, for which the resulting sensitivity values are stable.
  A sensitivity value for verification use should be selected based on this activity range.

Once this procedure has been established, regular quality controls of the system’s SPECT/CT sensitivity may be performed using the latter phantom geometry, adopting one single source strength within the identified activity range and making use of average projection count rates to make DTPU corrections. If deviations from the verification value are larger than a selected acceptance value, manifested in repeated controls, a new calibration measurement would be required. This measurement could then be executed using a homogeneous Jaszczak phantom with a ^131^I content in the range proven to provide robust results in step 1 above.

Among the advantages of the control routines under step 2 is that the full ^131^I dosimetry procedure is subject for verification, and any significant changes in properties of the system or the analysis software would be detected. Another advantage is that the Jaszczak phantom may easily be made available for other purposes by removing the spherical insert, despite using relatively long-lived ^131^I.

### Implications for clinical dosimetry

The aim of this study was to identify robust routines for reliable ^131^I calibration of SPECT/CT systems, being of importance for dosimetry. Despite the similar hardware of the two systems under study, minor differences in properties were identified. Such differences must be expected due to minor differences in collimators and detector crystals, emphasising the importance of establishing separate calibration factors for each system at a clinic. In this study, the system sensitivity difference was 5% when adopting Full-FOV SPECT/CT data from the homogeneous Jaszczak phantom, being in fair agreement with the 4% difference obtained for planar Full-FOV point-source data. However, one may argue that data from the detector periphery may be more prone to errors, while also being less relevant for clinical dosimetry. Adopting the concentration method, using data only from the central parts of the detectors, the system sensitivity difference obtained for the homogeneous Jaszczak phantom was < 1%, like the difference for planar data limited to a 13.2-diameter circle centred on the point source used. Possible reasons for the higher sensitivity of system (1) when including the detector periphery are unexpected contributions from peripheral parts of the detectors or more septum penetration. However, it was argued that the concentration method is more representative for dosimetry. This opinion was supported by the results for the 6-cm spherical Jaszczak insert; the delineation method (being recommended in guidelines [2]) returned < 1% system difference, while a 5% difference was obtained for Full-FOV SPECT/CT data.

The use of an adequate count-loss correction scheme was found to be crucial. Furthermore, calibration and verification of calibration should be performed using ^131^I phantom activity in a range exhibiting stable sensitivity values, which was between 250 and 1500 MBq for the systems in this study. At higher activities the count-loss corrections were unreliable, while at lower activities deviating results were encountered. Although robust calibration may be executed using ^131^I activity in the selected range, mitigating deviations at lower activities may be of importance in dosimetry. However, the deviations at low activities were relatively small (< 5%) when performing delineation analyses of images recorded from the 6 cm Jaszczak sphere, which is the object and type of analysis of highest relevance for dosimetry. Still, further studies are recommended to identify the underlying reasons for these deviations and evaluate their significance for dosimetry.

### Outlook

In any SPECT data recording, count rates will vary between projections, and accurate accounting of count losses would require a separate correction for each projection. This capability is currently not available, leaving the general user with the alternative to make overall corrections based on averages. Considering the shape of the DTPU correction curves in Fig. 4, using the average count rate over all projections will result in a smaller overall DTPU correction than separate projection corrections would render. This underestimation amplifies with higher count rates and larger variations between projections. In a calibration measurement, this error can be minimised by using an object rendering small variations in projection count rates. However, in a clinical patient examination, falling beyond scope of this study, one must expect a highly inhomogeneous source concentration and thus large variations in projection count rates, making the error from using the average projection count rate more severe.

Considering the increasing adoption of therapeutic dosimetry in nuclear medicine, eliminating any systematic errors in quantitative data is highly desirable. Accordingly, the development of systems with user-friendly procedures for making DTPU corrections on individual projections would be beneficial. The development of Cadmium-Zink-Telluride- based (CZT) systems, which can manage much higher count rates than scintillator-based systems, may reduce the need for such corrections. However, the availability of CZT systems with capability to measure high-energy gamma radiation, such as the 364.5-keV peak of ^131^I, is currently very limited. While awaiting the broader availability of these advanced CZT systems, vendor development of adequate user-friendly procedures for DTPU corrections of individual projections would be warmly welcome.

## Conclusions

The data from this study demonstrate that effective count-rate management is essential for achieving reliable results in system calibration for ^131^I SPECT/CT dosimetry. A range of 250 to 1500 MBq for the ^131^I phantom activity was found to produce count rates that offer robust calibration for the studied systems. A sensitivity calibration procedure with activity-concentration measurements in a homogenous Jaszczak phantom could subsequently be validated regularly using delineation analyses of a 6 cm spherical Jaszczak insert.

The collection of phantom data over a large range of ^131^I activities illustrated severe deviations in the absence of count-loss corrections. However, the well-controlled relative ^131^I activities in this study, being governed by physical decay only, also allowed for a loss correction scheme to be developed. It is argued that such a correction scheme should be based on total spectrum count rate, not the peak count rate, as the total count rate is the main driving force for these losses. In the absence of tools for correcting individual projections, the average total projection count rate may be used.

## Data Availability

Data is available on request.

## Footnotes

1 Leonid Tsukerman.

## Abbreviations

AC: Attenuation correction
CF: Calibration factor
CZT: Cadmium-zink-telluride, a material used in gamma-ray detectors
DTPU: Dead time and pulse pileup
EM: Emission
FOV: Field of view
GE: General Electric
HEGP: High energy general purpose (collimator)
IR: Iterative reconstruction
IRACSCRR: Iterative reconstruction with attenuation correction, scattering correction and resolution recovery
IRACSC: Iterative reconstruction with attenuation correction and scattering correction
OSEM: Ordered subset expectation maximisation
RR: Resolution recovery
SC: Scatter correction
TEW: Triple-energy window
